# A Case Report of Rapunzel Syndrome in 14-Year-Old Girl

**DOI:** 10.7759/cureus.74446

**Published:** 2024-11-25

**Authors:** Petar Stamov, Aleksandar Zlatarov, Stefan Mihaylov

**Affiliations:** 1 Department of General and Operative Surgery, Medical University "Prof. Dr. Paraskev Stoyanov", Varna, BGR

**Keywords:** abdominal pain, mental disorders, pediatric surgery, rapunzel syndrome, trichobezoar, trichophagia

## Abstract

Trichobezoar or hairball in the proximal part of the gastrointestinal tract is a rare condition that occurs mainly in young and adolescent females. Since human hair is resistant to digestive enzymes and resistant to peristalsis, it easily accumulates between the folds of the mucosa. Over time, food and mucus accumulate within the hair, forming a compact mass that fills almost the entire lumen. When a trichobezoar passes through the pylorus and reaches the duodenum, and in some cases even the jejunum, it is referred to as Rapunzel syndrome. The treatment is surgical. Depending on the location and size of the trichobezoar, it can be removed endoscopically, laparoscopically, or by conventional laparotomy. Our report aims to present the surgical treatment of Rapunzel syndrome, as part of a comprehensive approach to this disease. We present a 14-year-old girl who was diagnosed and treated in our department. According to the world practice and our experience, conventional laparotomy as a method of treatment remains the first choice. The smaller gastrointestinal trichobezoars are asymptomatic or with unusual clinical manifestations. They grow undetected and combined with mental disorders can lead to significant gastrointestinal complications such as mainly intestinal obstruction and rarely erosions, ulcers, and perforation. The treatment is multimodal including surgeons, pediatricians, and psychiatrists.

## Introduction

Trichobezoars are formed as a result of ingestion of hair or hair-like fibers [1]. They are usually found in the stomach, but as their volume increases over time, they can spread to the small intestine. The condition in which the trichobezoars reach the small intestine is defined as Rapunzel syndrome [2,3].

The Rapunzel syndrome was first reported in the literature by Vaughan et al. in 1968 [4]. Trichobezoar or hairball in the proximal part of the gastrointestinal tract is a rare condition that occurs mainly in young and adolescent females. Human hair is resistant to digestive enzymes and peristaltic movement, allowing it to accumulate easily within the mucosal folds. Over time, food particles and mucus become trapped in the hair, gradually forming a dense mass that can occupy nearly the entire lumen. The initial symptoms of trichobezoar can be categorized as nonspecific. In general, rarely are bezoars associated with gastrointestinal complications such as intestinal perforation, peritonitis, steatorrhea, protein-loss enteropathy, pancreatitis, obstructive jaundice constipation, intussusception, appendicitis, and pneumatosis intestinalis. Treatment is surgical. Depending on the location and size of the trichobezoar, it can be removed endoscopically, laparoscopically, or by conventional laparotomy. This report presents a case of Rapunzel syndrome found in a 14-year-old patient with no significant previous illnesses.

## Case presentation

**Figure 1:**
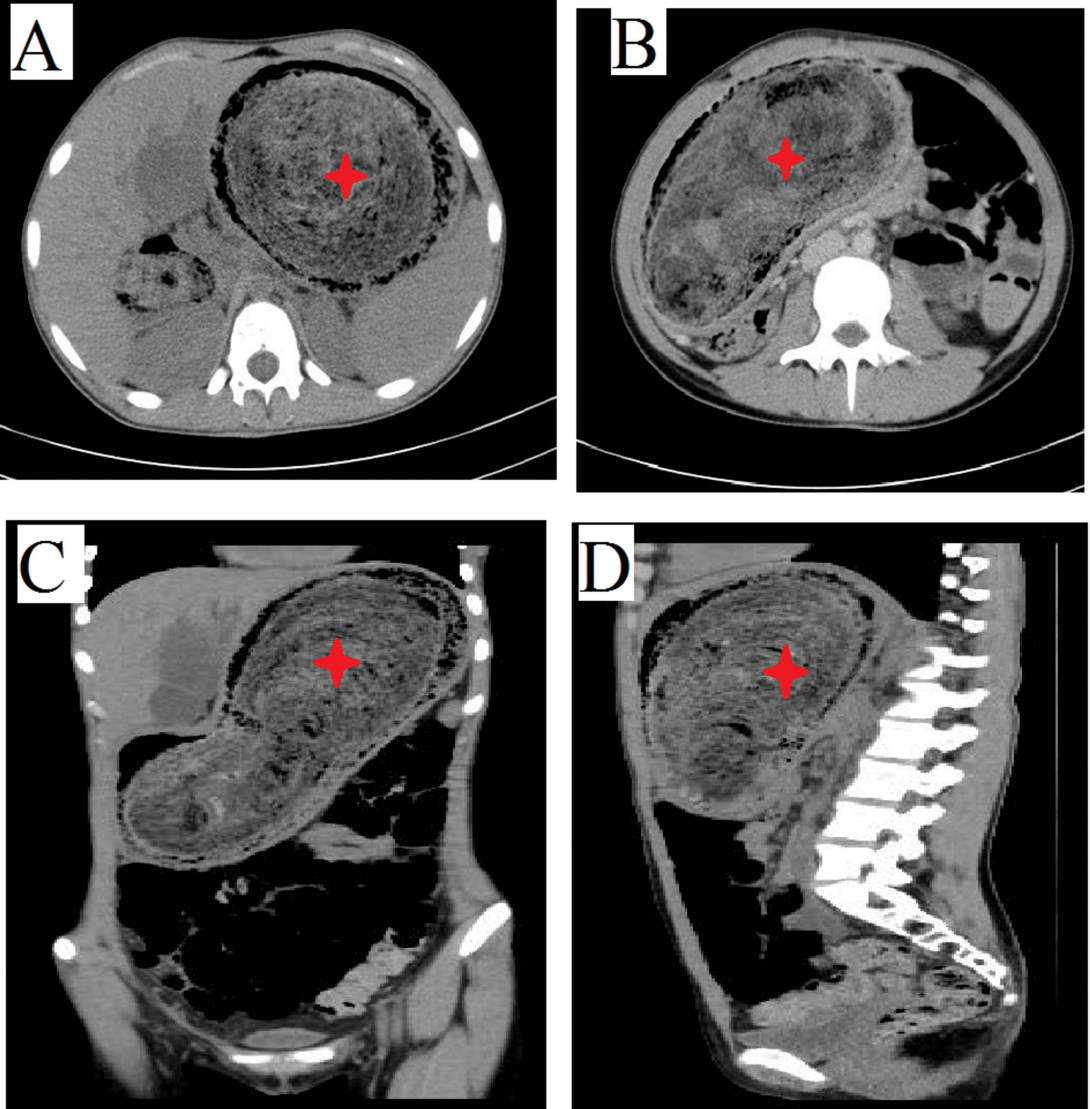
The abdominal computed tomography. It shows the stomach is distended and filled with heterogeneous mass in different views - axial (A, B), coronal (C), and sagittal (D).

In our case, we present a 14-year-old girl who for several months has had intermittent abdominal pain accompanied by repeated episodes of vomiting. The day before hospitalization, the pain intensified and she refused to take liquids and food. She was initially hospitalized at the Pediatric Department. The patient has been consulted with a pediatrician and due to the palpable mass in the abdomen and ineffective conservative treatment, the patient has been referred to the Department of Pediatric Surgery at St. Marina University Hospital. A CT scan of the abdomen was performed, which revealed a dilation of the stomach filled with inhomogeneous contents (Figure 1).

**Figure 2:**
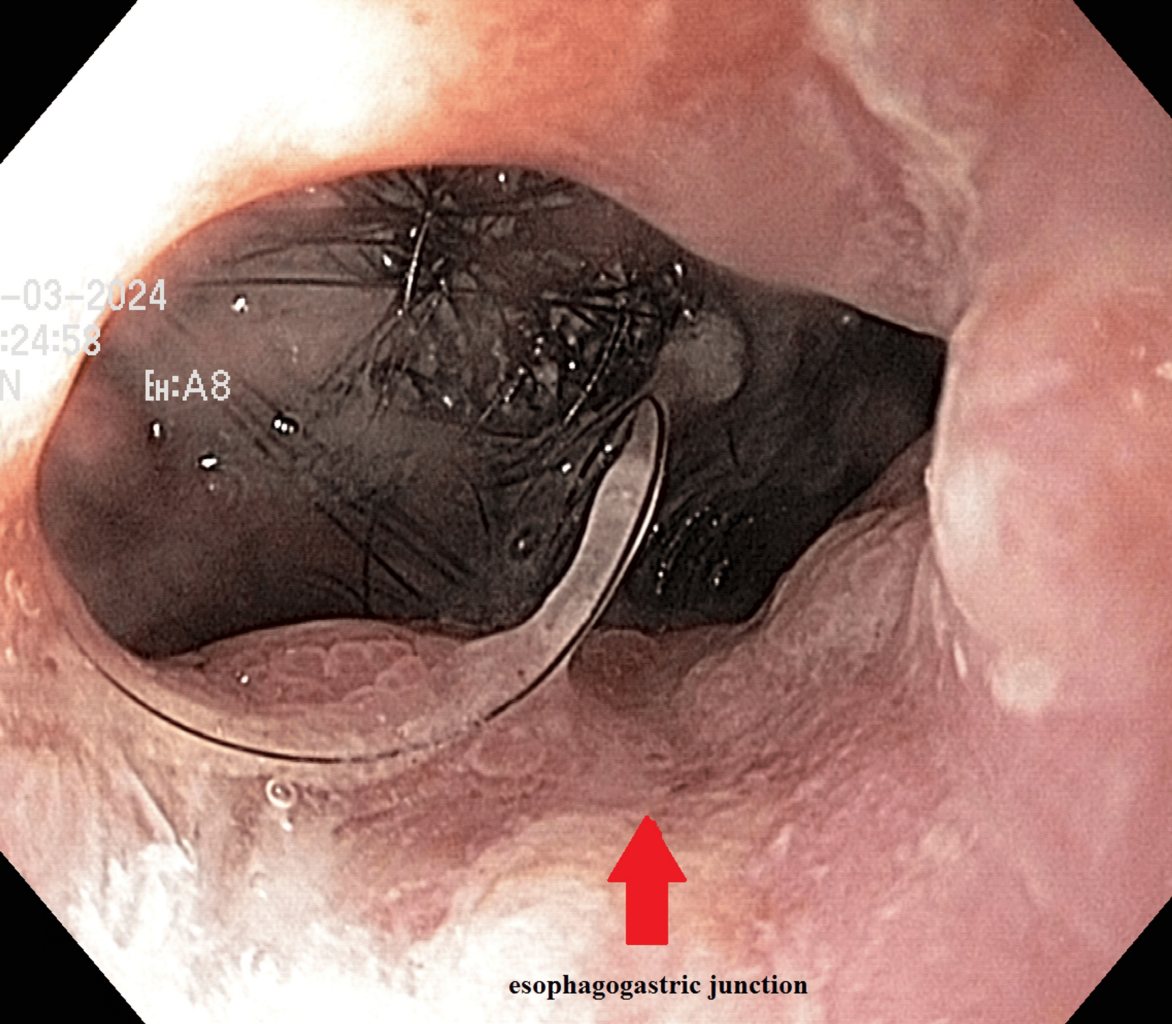
Upper gastrointestinal endoscopy. The aspect of trichobezoar at the level of the esophagogastric junction.

**Figure 3:**
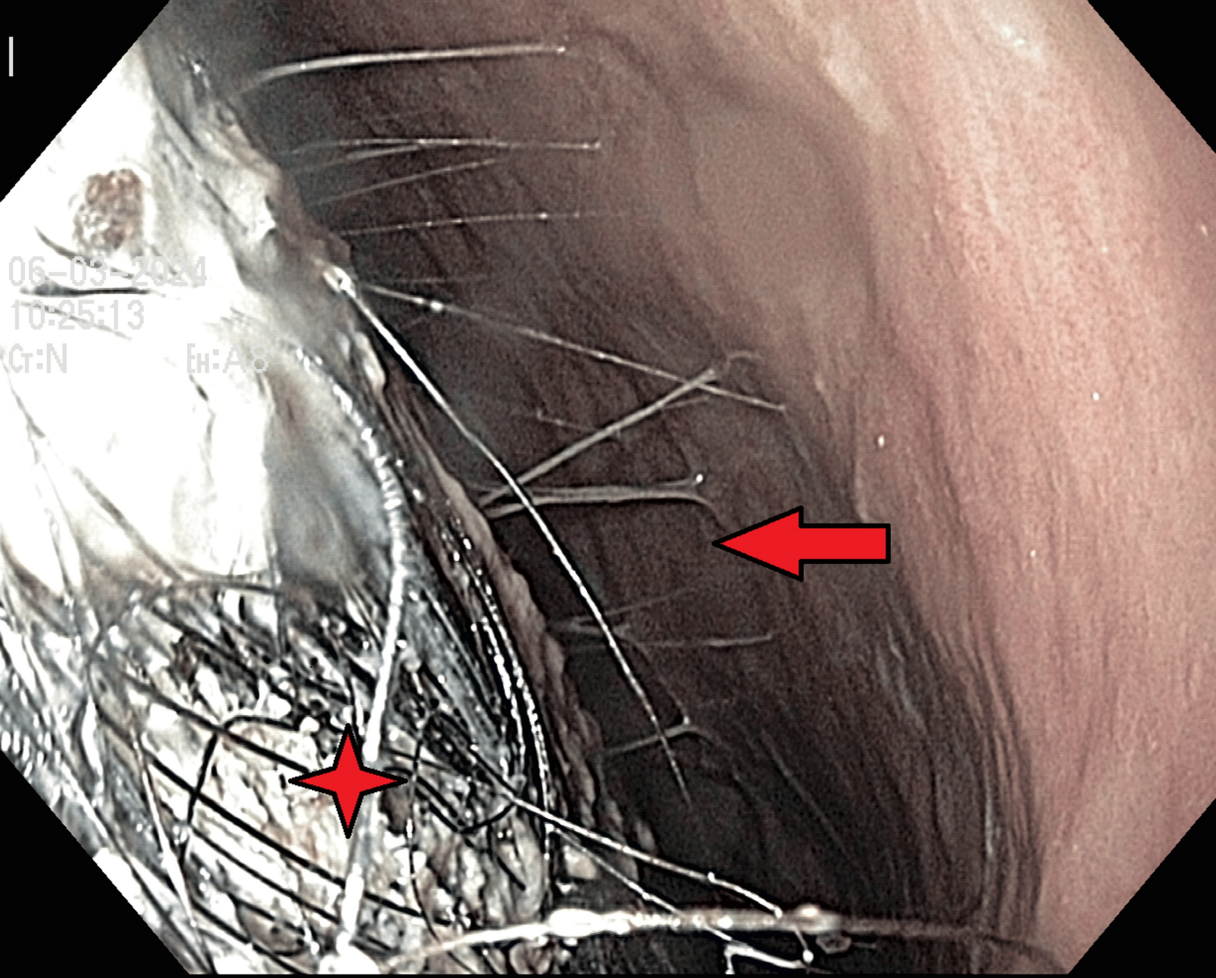
Gastric content on upper endoscopy. Visible trichobezoar(♦), completely obstructing the lumen (←) of the stomach.

The patient’s vital signs on admission to the pediatric surgery department were, respectively: respiratory rate 17 breaths/min, heart rate 87 beats/min, and blood pressure 110/60 mmHg. Local examination revealed a slightly painful abdomen in the epigastric and umbilical regions. A solid mass was palpated in the epigastric area and right hypochondrium along with localized tenderness. With a more detailed history, a psychological moment related to the adoption of a child into the family was established, after which the patient became “more closed” and, in fact, the complaints started from then. A detailed anamnesis revealed a psychological event related to the adoption of a child into the family four years ago, after which the patient became more withdrawn. The onset of complaints can be traced to that time. Over the past five months, the patient has lost approximately 5 kg. During a private conversation, the patient admitted to actively pulling out and swallowing her hair. A gastroscopy performed during hospitalization confirmed the presence of a trichobezoar in the stomach. Additionally, the clinical examination noted patchy hair loss on the scalp, which is consistent with trichotillomania. During hospitalization, gastroscopy was performed, which confirmed the presence of trichobezoar in the stomach (Figures 2-3).

**Figure 4:**
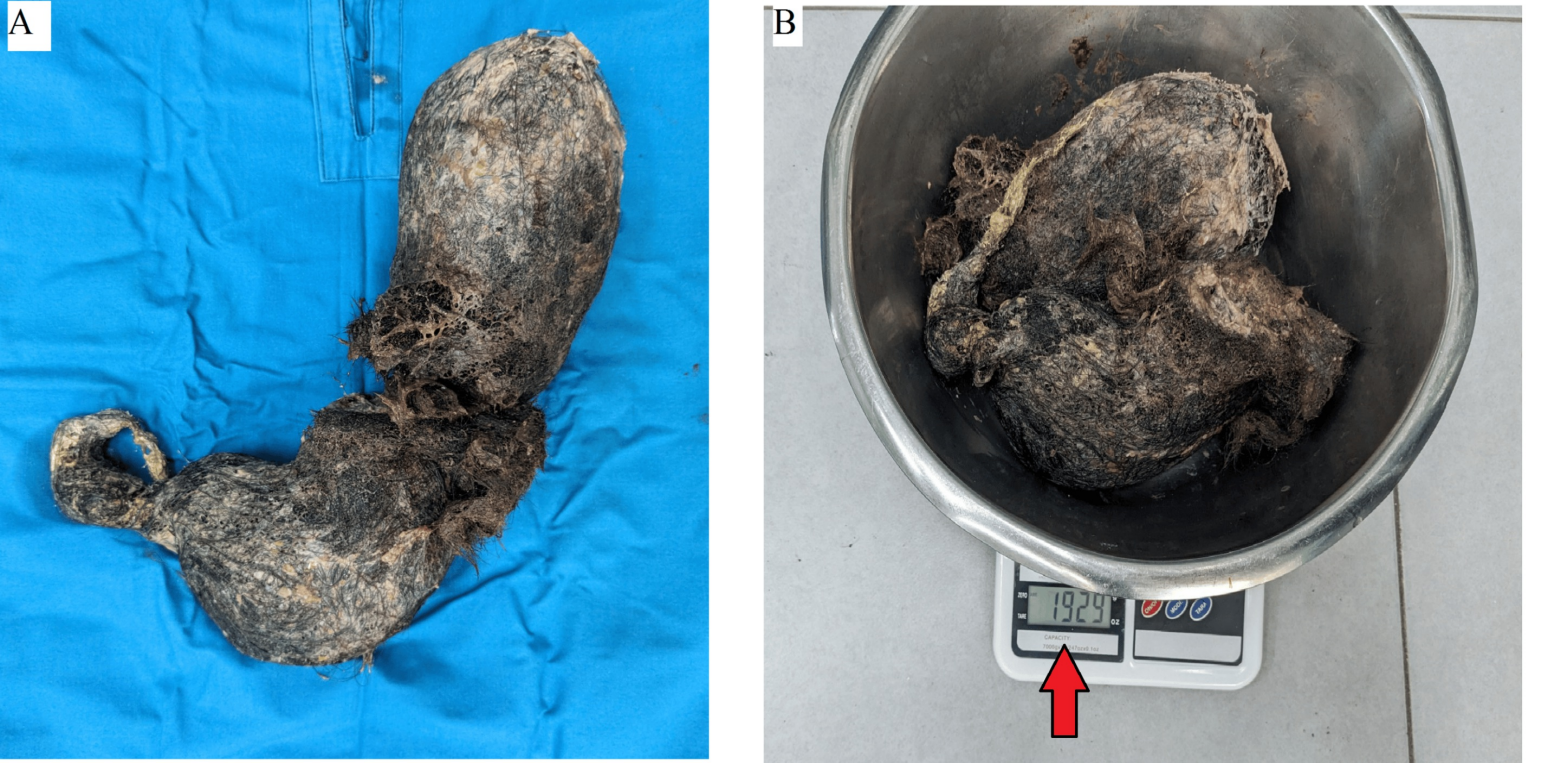
A macroscopic aspect of trichobezoar. A stomach-shaped trichobezoar with a tail to the pylorus and duodenum (A) and showing its weight of 1929 g (B).

Due to its large size and the inability to extract it endoscopically, a classic laparotomy was performed. Through a limited midline supraumbilical incision, we performed an anterior gastrotomy. A huge gastric trichobezoar and its tapering tail from the duodenum weighing 1929 g were completely removed without complications (Figure 4).

The postoperative hospital course was uneventful. The patient began oral intake of liquids on the third postoperative day and progressed to soft, semi-solid food on the fourth postoperative day. She was discharged on the seventh postoperative day. The patient was also assessed by a psychiatrist, and her parents reported an improvement in her behavioral symptoms.

## Discussion

The word “bezoar” comes from the Arabic “badzehr” or the Persian “padzhar”, both meaning “protection against poison”. Historically, bezoars, which are stone-like objects found in the intestines of certain animals, were believed to have antidotal properties and were used as remedies for poison. Today, bezoars are still used in some traditional medicine systems, including traditional Chinese medicine [5]. The term comes from the story of the long-haired princess Rapunzel, written in 1812 by the Brothers Grimm [6]. Due to the fact that human hair is resistant to digestive enzymes and resistant to peristalsis, it easily accumulates between the folds of the mucous membrane. Over time, remnants of food and mucus accumulate in the hairs, forming a compact mass that covers almost the entire lumen. In a large number of cases, the disease is accompanied by various mental disorders such as depression, obsessive-compulsive disorder, anorexia [7,8]. The most common bezoars in humans, and more specifically in adolescent girls with co-occurring neuropsychiatric disorders are trichobezoars. Although rare, cases in boys have also been described in the literature, with the youngest reported patient being a 3-year-old described by Hal et al. [9]. Of utmost importance for the diagnosis is careful anamnesis and a targeted search for the problem, because even in the presence of obvious alopecia, a large number of patients, including parents, refuse to provide this information. This is where the good communication skills and teamwork of the doctor with other specialists come in [10,11]. Clinically, the disease is usually asymptomatic until the trichobezoar grows significantly. Over time, various upper gastrointestinal symptoms appear, as well as fatigue and cachexia. Large trichobezoars are often visible and protrude through the anterior abdominal wall (Lamerton’s sign). Gastrointestinal perforation, diarrhea, obstruction, intussusception, anemia, and vitamin B12 deficiency are identified as the main complications of trichobezoar [12]. Imaging investigations such as ultrasound, barium swallow, and CT confirm the diagnosis. Upper esophagogastroscopy (EGS) is considered the gold standard in diagnosis, showing a mass formed by hair mixed with mucus and food, with a black color due to the effect of stomach acid on the hair protein [13]. Trichobezoar treatment should include the removal of the mass from the gastrointestinal tract and prevention of recurrence by treating the underlying physical or emotional cause. Endoscopic removal, although attractive, has a very low success rate, generally applicable to small trichobezoars [14]. Laparoscopy is a relatively better alternative compared to endoscopy but usually turns into an open laparotomy due to the sheer size of the mass. Surgical treatment is indicated in a large proportion of patients due to the clinical symptoms and the impossibility of removing it using alternative method such as endoscopic retrieval [15]. Surgical removal is performed by laparotomy and gastrotomy and is frequently the only feasible option in Rapunzel syndrome. Recently, treatment of trichobezoars with enzymatic preparations with an unsatisfactory effect has also been reported, mainly for smaller and early diagnosed bezoars.

## Conclusions

Rapunzel syndrome is a rare condition, most often seen in teenage girls with underlying mental health disorders. Smaller trichobezoars are typically asymptomatic but can grow unnoticed, leading to serious gastrointestinal complications like obstruction, ulcers, or perforations. In this case, the presence of a trichobezoar, combined with alopecia and suspected mental health issues, highlights the need to consider it in the differential diagnosis for nonspecific abdominal pain. Treatment involves a multidisciplinary approach, including surgical removal of the bezoar, psychological support, and prevention of recurrence.
